# A cross-sectional survey to estimate the prevalence of family history of colorectal, breast and ovarian cancer in a Scottish general practice population

**DOI:** 10.1038/sj.bjc.6602155

**Published:** 2004-09-21

**Authors:** E Wallace, A Hinds, H Campbell, J Mackay, R Cetnarskyj, M E M Porteous

**Affiliations:** 1 Public Health Department, Fife NHS Board, Springfield House, Cupar, Fife, Scotland; 2 Community Health Sciences, University of Edinburgh, Teviot Place, Edinburgh, Scotland; 3 The Genetics Unit, Institute of Child Health, 30 Guilford St, London WC1N1EH, UK; 4 South East Scotland Genetic Service, Western General Hospital, Crewe Road, South Edinburgh, EH4 2XU, Scotland

**Keywords:** cross-sectional, family cancer history, genetic risk, prevalence

## Abstract

A cross-sectional survey of all patients aged 30–65 in four general practices within one Local Health Care Co-operative in Fife, Scotland was undertaken to measure the prevalence of family history of colorectal, breast and ovarian cancer. A total of 7619 patients aged 30–65 responded to a postal questionnaire (response rate 59%). In all, 17% of respondents (1324, 95% Cl 16–18%) reported a relative affected by colorectal, breast or ovarian cancer. Of those, 6% (78, 95% CI 5–7%) met the Scottish guidelines for referral for genetics counselling. In all, 2% (24, 95% CI 1–3%) of all individuals with an affected relative had received genetic counselling and risk assessment. Of these, 25% (6, 95% CI 8–42%) met the moderate- or high-risk criteria for developing a cancer. In conclusion, the number of patients who are at a significantly increased risk of cancer on the basis of a family history is small (approximately 10 per General Practitioner (GP) list). It is therefore unrealistic to expect GPs to develop expertise in genetic risk estimation. A simple family history chart or pedigree is one way that a GP can, within the constraints of a GP consultation, determine which patients should be reassured and which referred to the local cancer genetic clinic.

Cancer is one of the three health priorities of the National Health Service in Scotland (NHSiS) (Scottish Office, 1998). Local Health Care Co-operatives (LHCCs) were created in Scotland in 1998 to provide local management of services, and are made up of representatives of local general practices and local service groups and patient groups (Scottish Office, 1998). They have been charged with measuring health needs within their communities to reflect the clinical priorities for the area and to support the development of population-wide approaches to health improvement and disease prevention (Scottish Office, 1997).

Cancer genetics is the fastest growing area of clinical genetics (Wonderling *et al*, 2001). In Scotland, the four Regional Genetic Centres co-ordinate accurate risk assessment to ensure that individuals referred for screening investigations such as mammography and colonoscopy fulfil the national criteria laid down by the Cancer Genetic subgroup of the Scottish Cancer Group (Table 1

**Table 1:** The Cancer Genetic Sub-committee family history criteria for enrolment in a screening programme for breast, ovarian or colorectal cancer

*Breast*
Moderate risk
One first-degree relative with bilateral breast cancer
One first-degree relative with breast cancer diagnosed under age 40 years or one first-degree male relative with breast cancer diagnosed at any age
Two first- or first- and second-degree relatives with breast cancer diagnosed under age 60 years and/or ovarian cancer at any age on the same side of the family
Three first- or second-degree relatives with breast or ovarian cancer on the same side of the family (always one first-degree relative unless history is via father)
High risk
An individual with BRCA1 or BRCA2 mutations or other known predisposing gene mutations or the untested first-degree relative of a mutation carrier
One first-degree relative (or second-degree relative via intervening male relative) in a family with four or more relatives affected with breast cancer or ovarian cancer in three generations
One first-degree relative (or second degree via father) with breast and ovarian cancer
*Ovarian*
Moderate risk
Two or more first- or first- and second-degree relatives with ovarian cancer
Two first- or first- and second-degree relatives with ovarian cancer at any age and breast cancer diagnosed under 50 years
One ovarian cancer and two breast cancers diagnosed less than 60 years on the same side of the family in first-degree relatives or second degree via a male
Two first- or second-degree relatives with colorectal cancer and/or endometrial cancer and one with ovarian cancer one affected relative with ovarian cancer and HNPCC family history
High risk
An individual with BRCA1 or BRCA2 mutations or other known predisposing gene mutations or her untested female relatives.
First-degree relative with breast and ovarian cancer
*Bowel*
Moderate risk
One first-degree relative with colorectal cancer under age 45 years
Two individuals affected with colorectal cancer (one less than 55 years) who are first-degree relatives of each other and one a first-degree relative of the consultant
Three affected family members with colorectal or endometrial cancer who are first-degree relatives of each other and one a first-degree relative of the consultant
High risk
An individual with a mutation in one of the mismatch repair genes or their untested first-degree relatives
A family history compatible with HNPCC according to Amsterdam or modified Amsterdam criteria

Individuals are judged to be at low risk if their family history does not meet the moderate risk criteria for screening.

). The lifetime risks for breast, colon and ovarian cancers in the general population are approximately one in 10, one in 60 and one in 90, respectively (ISD, 1998). All general practitioners (GPs) will, therefore, have patients with a relative with one of these cancers. An unknown proportion of these patients are likely to seek counselling and advice regarding their risk of developing cancer (Biesecker *et al*, 1993). The relative risk associated with a family history of these cancers has been widely reported (St John *et al*, 1993; Slattery and Kerber, 1994; Pharoah *et al*, 1997). The challenge is to identify the minority at significantly increased genetic risk of developing cancer while reassuring the majority whose family history does not indicate a likely increased cancer risk above that of the general population.

A major problem in planning cancer genetic services is that it is not known as to what proportion of the population fit into the various cancer genetic risk categories. The Scottish Office report ‘Cancer Genetics Services in Scotland’ (Haites, 2000) recognised that ‘at present there is no means of identifying the total population who have a family history which places them at a significantly increased risk of developing breast, colorectal or ovarian cancer’. The report also noted that the uncertainty of these estimates makes it impossible to predict future costs for the provision of a risk estimation and screening service.

Risk estimation is based on the number of affected individuals within the family, the pattern of cancers and the age of onset of cancer. It is therefore necessary for the clinician to take a careful family history. This process is time-consuming and many GPs are unsure of their ability to obtain an accurate family tree and assess genetic risk (Fry *et al*, 1999). Pre-clinical family history questionnaires have been used extensively by genetic departments. This study was designed to evaluate how a similar questionnaire would be addressed by a general practice population and whether such a questionnaire might provide data in a form to facilitate GP cancer genetic risk estimation.

We report the results of a cross-sectional survey conducted between May 1999 and October 2000 of patients in General Practice aged between 30 and 65 years to assess the prevalence of a significant family history of colorectal, breast, or ovarian cancer and to identify the number of individuals with a family history that had been referred onto the Clinical Genetic Service. Ethical approval for the study was granted by the Fife Local Research Ethics Committee.

## PARTICIPANTS AND METHODS

A postal survey of all patients aged 30–65 years from four general medical practices covering over 99% of the population within one LHCC in Fife, Scotland was undertaken using a cancer family history questionnaire that had been developed and evaluated by a Cambridge-based research team (Leggat *et al*, 1999). The questionnaire was adapted to determine whether the patients had any concerns regarding their own risk of developing cancer and, if so, whether they had ever been referred to a cancer genetic specialist or had received any form of genetic counselling (questionnaire available online at http://137.195.14.43/cgi-bin/W ebObjects/genisys.woa/wa/showD oc?docid=208).

Patients were asked if they had any family members (grandparents, aunts, uncles, father, mother, brothers, sisters and children) who had had colorectal, breast or ovarian cancer and the age at which these cancers were diagnosed. Those with no affected relatives were requested to return the questionnaire at this point. Those with a family member affected were asked to complete a detailed family history including relationship to the affected individual, site of cancer and age at and date of diagnosis. In all, 305 randomly selected participants reporting a family history of cancer (23% of total) were interviewed by telephone (*n*=254) or in person by a genetic nurse (*n*=51) to check the consistency of the information collected via the postal survey. A fieldworker telephoned 101 of those reporting no family history to confirm that there was no family history of colorectal breast or ovarian cancer in their families.

## RESULTS

**Figure 1:**
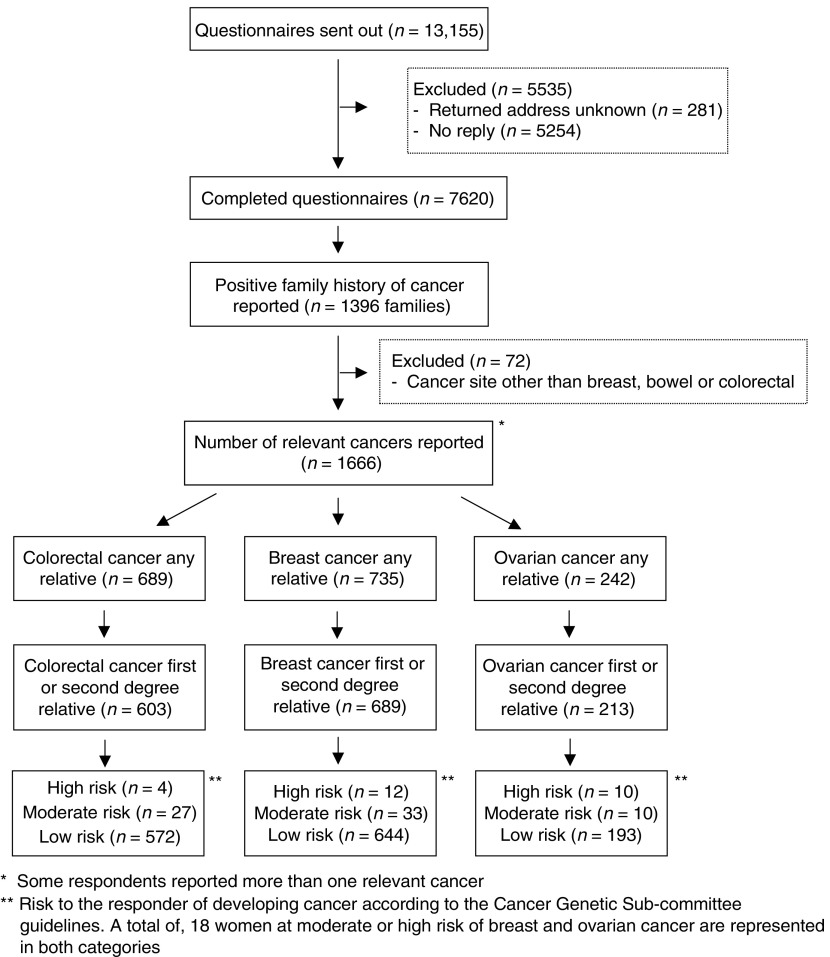
Flow diagram of response and results of a survey to estimate the prevalence of a family history of selected cancers in a Scottish population surveyed in 1999–2000.

A total of 13 155 questionnaires were mailed, of which 5535 were excluded from the study; 281 were returned address unknown and 5254 were not returned by the patient. In all, 7620 (3386 males, 4234 females) were completed and returned (Figure 1

). The overall response rate was 59%. A total of 1396 (18%, 95% CI 17–19%) responders reported a family history of cancer. When checked by a genetics nurse, 72 questionnaires reported relatives with a history of cancers at other sites and were excluded from any further analysis. In all, 17% of respondents (1324, 95% Cl 16–18%) therefore identified themselves as having a history of colorectal, breast, or ovarian cancer in a first- or second-degree relative. Of these, 918 were females and 375 males. Some respondents reported a family history of more than one of these cancers. In total, 78 respondents with a family history were classified as being at medium or high risk of developing colorectal, breast, or ovarian cancer, and thus met the guidelines for referral to cancer genetics services in Scotland for risk assessment (Haites, 2000). This represents approximately 6% (95% CI 5–7%) of all respondents reporting a family history of cancer.

### Colorectal cancer

In all, 31 respondents reporting a family history of colorectal cancer met the national guidelines for referral for risk assessment, 11 males and 20 females, that is, 5% (95% CI 3–7%) of those reporting a family history of colorectal cancer and 2% (95% CI 1–3%) reporting a history of any of the three cancers or 0.41% (95% CI 0.26–0.55%) of the population surveyed.

### Breast cancer

In all, 27 of the female respondents met national guidelines for referral for risk assessment for breast cancer only, that is, 3% (95% CI 2–4%) of all female respondents reporting a family history of cancer or 0.64% (95% CI 0.40–0.88%) of the total female population surveyed.

### Ovarian cancer

Two female respondents met the national guidelines for referral for risk assessment for ovarian cancer only, that is, 0.2% (95% CI 0–0.5%) of all females reporting a family history of cancer or 0.05% (95% CI 0–0.1%) of the total female population surveyed.

### Breast and ovarian cancer

In all, 18 female respondents met the national guidelines for referral for risk assessment, that is, 2% (95% CI 1–3%) of all female respondents reporting a family history of cancer or 0.43% (95% CI 0.22–0.62%) of the total female population surveyed.

### Interviews of re-contacted participants

A validation study was undertaken in order to assess the consistency of this information. In all, 352 patients reporting a family history of cancer were randomly selected and asked to discuss their history with a genetic nurse either face to face or by telephone. Of these, 305 (87%) responded and their family history was verbally confirmed. Of these, 17 (6%, 95% CI 3–8%) were assessed to be at a moderate to high risk of developing colorectal cancer and thus met the national criteria for referral for risk assessment, 28 (9%, 98% CI 6–12%) met the referral criteria for breast cancer and three (1%, 95% CI 0–2%) for ovarian cancer.

As a result of this group being interviewed by the genetic nurse, the risk of 21 (7%, 95% CI 4–10%) of the respondents was altered. The estimated risk of one or more of the three cancers was increased for 16 of the respondents, although in six cases it was difficult to verify the risk due to incomplete information, for example, age of diagnosis of cancer in relative. For five respondents, the estimated risk of cancer was reduced. Only four (4%, 95% CI 0.2–8%) of the 101 respondents who originally reported no family history of breast, ovarian or colorectal cancer in the family history form subsequently mentioned a family history on interview with a fieldworker. All four were assessed to be at low risk of developing cancer.

### Contact with health services

In all, 15% of respondents who reported a positive family history of these cancers had discussed their concerns with their GP, the great majority during the last 3 years. Out of these respondents, 86% (and 87% of the 30 respondents found to be at moderate or high risk) had raised the issue themselves (rather than their GP asking them about the family history of cancer). In all, 10% of respondents reporting any family history of cancer (and 22% of those at moderate/high risk) had been referred to a specialist to discuss their risk of cancer and 2% (25% of those at moderate/high risk) had received genetic counselling in the past.

### Workload implications for GPs and cancer genetics clinics

Using these results to estimate workload for GPs and cancer genetic clinics in the rest of Scotland, the following figures are obtained:


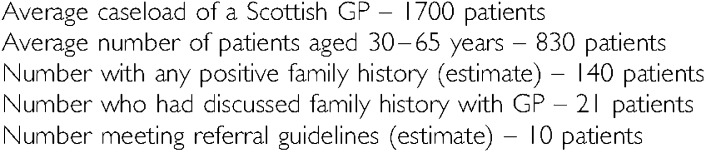


Potentially, only one in 14 patients attending a GP with a positive family history of cancer needs to be referred to regional cancer genetics services for further risk assessment.

## DISCUSSION

A valid response rate of 59% was achieved for the postal questionnaire used in this study. This is considerably higher than that in a previously published study using a similar questionnaire where the response rate was 29% (Leggat *et al*, 1999). Possible explanations for this high response rate to the questionnaire include: the study was led by the principal GP of one of the participating general practices and was thus well known to most patients; a press release publicising the study was issued prior to mailing; one reminder was sent out to nonresponders 2 weeks after mailing the questionnaire; and a colorectal cancer screening study had recently been undertaken in one of the four participating GP practices. When compared with the nonresponders, the responders were significantly older (mean age 48 years *vs* 44 years), similar to that reported in the previous study which evaluated the family history of cancer questionnaire.^9^ The study made no allowance for multiple sampling of the same family, but the aim was to assess the burden of cancer genetics in a GP practice. Males with a family history of breast or ovarian cancer were assessed as low risk, as no clinical screening is indicated for them.

We have recently shown in Scotland that such reports of a positive family history of cancer are rarely incorrect but may substantially underestimate the true prevalence of a history of cancer in relatives, especially among second-degree relatives, when compared to cancer registry records (Mitchell *et al*, 2004). We only attempted to ‘validate’ a sample of positive reports of family history of cancer in this study. It is likely that a study which also involved an analysis of cancer registry records of all relatives would yield a higher estimate for the family history of cancer. Thus, the prevalence of family history of cancer in this study can be considered to represent a minimum estimate. Nevertheless, patients make decisions about seeking advice about their cancer risk based on their family history as they perceive it and so that data presented in this report are important in seeking to plan services for these patients.

It is interesting to note the higher incidence of moderate- or high-risk family histories in the subgroup of participants that agreed to be interviewed. This may reflect a greater interest in discussing their situation in the moderate- and high-risk groups.

Prior to the study, it was anticipated that some respondents might experience anxiety concerning their own risk of cancer as a result of completing the family history questionnaire. Participants were invited to voice their anxieties by phone with the study team who could then arrange an appointment with a genetic nurse. However, it was only necessary for the genetic nurse to contact two respondents in relation to this issue and she was able to provide advice and reassurance in both cases. Discussion with GPs in the practices involved revealed no contact with patients worried by the results of the study. Many of the respondents did admit to worries about their family history when interviewed, but had not taken advantage of genetic counselling. In fact, those at the greatest risk were the ones who reported least use of the service.

The majority of questionnaires were completed correctly and many respondents included a great deal of information about their family history of cancer, sometimes involving obtaining details from family members living abroad. For GPs faced with patients consulting with concerns about their family history, a suitable response would therefore be to ask the patient to complete a similar family history form and to rely on this in making a decision as to whether or not to refer the patient to the local cancer genetics clinic.

Cancer genetics referral guidelines are quite complex. Therefore, computer programmes have been developed based on referral guidelines to support the decision-making by GPs. However, as GPs will see only a few patients a year, acquiring all of the skills necessary for genetic counselling or to operate such programmes may be unlikely to be accorded a high priority. In addition, newly acquired skills following training are likely to degrade over time without frequent reinforcement. We suggest that GPs could use a questionnaire to collect information and then pass it on to the local genetic nurse, primary care genetic clinician or cancer centre for a rapid assessment as to whether further action should be taken.

The number of patients seeking genetic counselling has increased sharply over the last few years (Wonderling *et al*, 2001). This study has shown that only about one in 14 patients attending a GP with a positive family history of cancer needs to be referred to regional cancer genetics services for further risk assessment. The importance of the gate-keeping role of the GP is likely to increase in future. Our experience gained during the course of this study suggests that this role might be facilitated by the use of a self-completion family history form in general practice. Information collected by this means tallies closely with that obtained from interviews with trained genetic nurses and permit accurate risk assessments which can guide referral decisions.
